# Cost analysis of an integrated disease surveillance and response system: case of Burkina Faso, Eritrea, and Mali

**DOI:** 10.1186/1478-7547-7-1

**Published:** 2009-01-08

**Authors:** Zana C Somda, Martin I Meltzer, Helen N Perry, Nancy E Messonnier, Usman Abdulmumini, Goitom Mebrahtu, Massambou Sacko, Kandioura Touré, Salimata Ouédraogo Ki, Tuoyo Okorosobo, Wondimagegnehu Alemu, Idrissa Sow

**Affiliations:** 1 Centers for Disease Control and Prevention, Atlanta, Georgia, USA; 2 WHO Country Office, Asmara, Eritrea; 3 Disease Prevention and Control, Ministry of Health, Eritrea; 4 WHO Country Office, Bamako, Mali; 5 Service Surveillance des Maladies, Ministère de la Santé, Mali; 6 Direction des Etudes et de la Planification, Ministère de la Santé, Ouagadougou, Burkina Faso; 7 WHO African Regional Office, Harare, Zimbabwe

## Abstract

**Background:**

Communicable diseases are the leading causes of illness, deaths, and disability in sub-Saharan Africa. To address these threats, countries within the World Health Organization (WHO) African region adopted a regional strategy called Integrated Disease Surveillance and Response (IDSR). This strategy calls for streamlining resources, tools, and approaches to better detect and respond to the region's priority communicable disease. The purpose of this study was to analyze the incremental costs of establishing and subsequently operating activities for detection and response to the priority diseases under the IDSR.

**Methods:**

We collected cost data for IDSR activities at central, regional, district, and primary health care center levels from Burkina Faso, Eritrea, and Mali, countries where IDSR is being fully implemented. These cost data included personnel, transportation items, office consumable goods, media campaigns, laboratory and response materials and supplies, and annual depreciation of buildings, equipment, and vehicles.

**Results:**

Over the period studied (2002–2005), the average cost to implement the IDSR program in Eritrea was $0.16 per capita, $0.04 in Burkina Faso and $0.02 in Mali. In each country, the mean annual cost of IDSR was dependent on the health structure level, ranging from $35,899 to $69,920 at the region level, $10,790 to $13,941 at the district level, and $1,181 to $1,240 at the primary health care center level. The proportions spent on each IDSR activity varied due to demand for special items (e.g., equipment, supplies, drugs and vaccines), service availability, distance, and the epidemiological profile of the country.

**Conclusion:**

This study demonstrates that the IDSR strategy can be considered a low cost public health system although the benefits have yet to be quantified. These data can also be used in future studies of the cost-effectiveness of IDSR.

## Background

Communicable diseases remain the most common causes of death, illness and disability in African countries. Lopez et al. (2006) reported that one-third of the deaths in low-and-middle income countries in 2001 were from communicable and parasitic diseases and maternal and nutritional conditions [1,2]. In addition, the economic cost in terms of prevention, treatment, and loss of productivity is enormous [3-5]. Although a number of studies on economic evaluation of interventions against communicable diseases have been reported in the literature [6,7], most of these studies in sub-Saharan Africa have focused on individual disease-specific intervention programs, such as prevention or treatment of malaria, measles, meningitis, tuberculosis and HIV/AIDS [5,8-15]. Relatively few studies have looked at the economics of integrating resources for disease surveillance and public health response activities [16].

**Figure 1:**
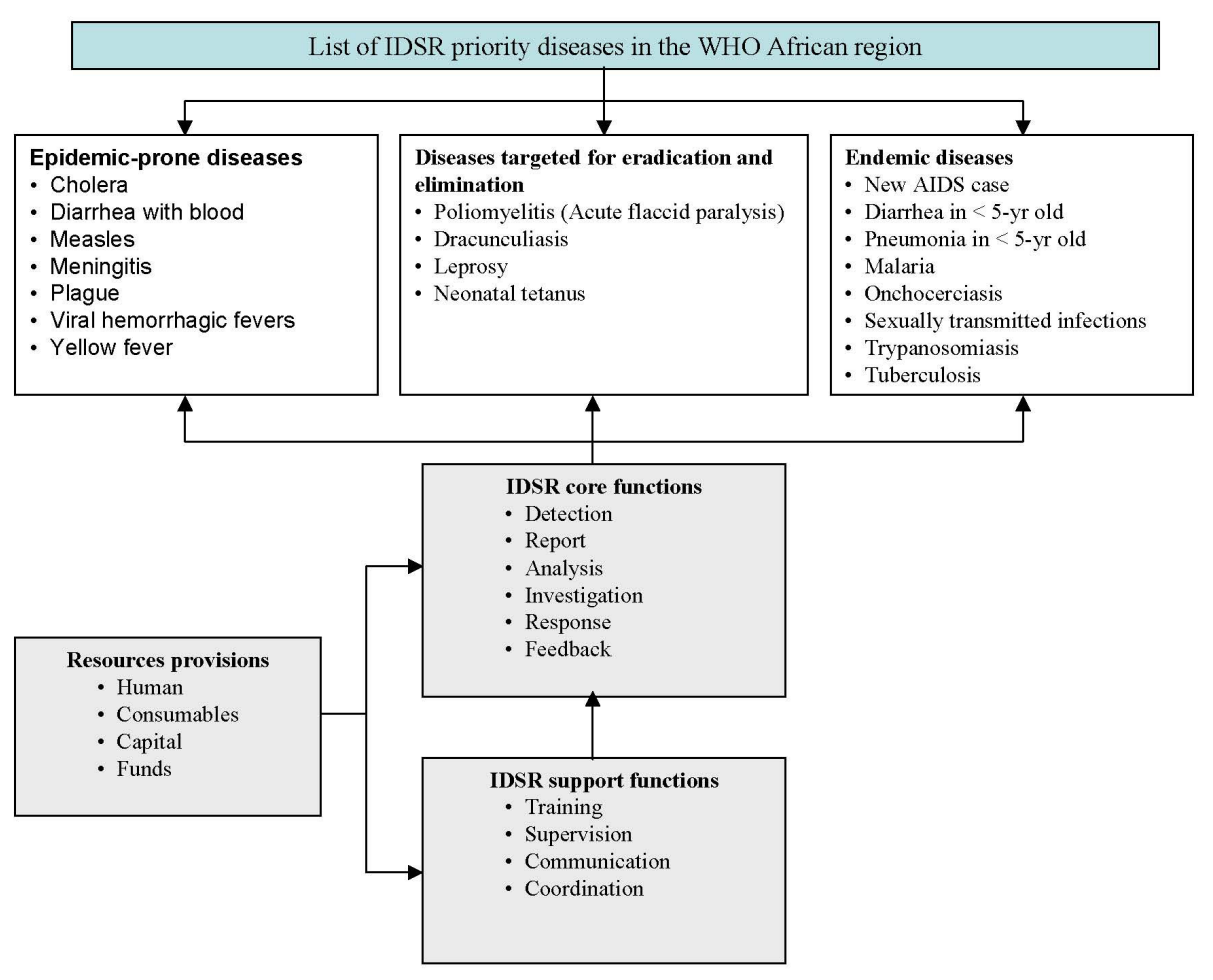
**Recommended IDSR priority diseases, core functions and activities in the WHO African region**.

Surveillance is an important component of disease prevention and control programs. It is useful in early detection of unusual events for effective and timely action, monitoring and evaluation of interventions and guiding selection of appropriate corrective measures [17]. In 1998, the Regional Committee of the World Health Organization Africa region (WHO-AFRO) adopted a strategy called Integrated Disease Surveillance and Response (IDSR) [18]. Under the IDSR strategy, countries address improvements in infrastructure capacities and support activities and select a number of priority diseases and health risk conditions from a list of the 19 communicable diseases that affect African communities (Figure 1) [19-21]. By December 2007, considerable progress had been achieved, with 43 of the 46 countries having assessed their national surveillance system and developed plans of action; 41 countries had already adapted the technical guidelines to meet their own public health priorities and situations and then launched IDSR activities at their district levels; and 33 countries had trained staff on IDSR in at least 60% of their districts (Table 1).

**Table 1:** Progress with IDSR implementation in the WHO AFRO African Region†: 2001 – 2007

IDSR Activities	Number of countries (% of total 46 countries)						
	
	2001	2002	2003	2004	2005	2006	2007
Sensitization of Ministry of Health officials and stakeholders on IDSR	22 (48%)	35 (76%)	36 (78%)	43 (96%)	44 (96%)	44 (96%)	44 (96%)
Assessment of national surveillance and response, including laboratory	22 (48%)	35 (76%)	36 (78%)	43 (93%)	43 (93%)	43 (93%)	43 (93%)
Development of IDSR plans of action	13 (28%)	31 (67%)	32 (70%)	43 (93%)	43 (93%)	43 (93%)	43 (93%)
Adaptation of generic IDSR technical guidelines*	1 (2%)	26 (57%)	35 (76%)	39 (85%)	41 (89%)	41 (89%)	41 (89%)
Adaptation of generic IDSR training materials*		1 (2%)	20 (43%)	35 (76%)	39 (85%)	39 (85%)	39 (85%)
Training staff on IDSR in at least 60% of the districts							33 (72%)
Publishing feedback bulletins							32 (70%)

†Source: Progress with IDSR implementation .

*Materials were developed by WHO AFRO and the US Centers for Disease Control and Prevention (CDC)

In order to better understand the investment and implementation costs of this IDSR strategy, the IDSR multi-partner task force that guides the implementation of this regional strategy recommended that the partners undertake cost analyses and cost-effectiveness studies. Therefore, the purpose of this study was to analyze the incremental costs of establishing and subsequently operating activities for detection and response to the priority diseases under the IDSR.

## Methods

### Study countries

The study was conducted in Burkina Faso, Eritrea, and Mali, countries where infectious diseases such as cholera, malaria, meningococcal meningitis and yellow fever are either epidemic or endemic (Table 2). Burkina Faso, with a population of 13.2 million, is divided into 13 health regions, 55 districts and has more than 1,232 primary health care centers. Mali, with about 13.5 million inhabitants, has nine regions, 57 districts and over 709 fully operational primary health care centers. Eritrea, with an estimated population of 4.4 million, is divided into six regions, 57 districts and has 664 primary health care centers. These three countries were selected for this study because each had fully established IDSR leadership and structures at the national level by 2002, with implementation at regional and district levels in 2003 and 2004, respectively.

**Table 2:** Summary of country health status*

	Burkina Faso	Eritrea	Mali	WHO African Region†
Total population (× 1,000)	13,228	4,401	13,518	738,083
Total expenditure on health (as % of GDP)	5.6	4.4	4.8	
Adult mortality rate (per 1000 population)	441	313	452	492
Under-5 mortality rate (per 1000 live births)	192	82	219	167
Year of life lost by communicable diseases (%)	87	81	86	59
**Causes of death among children under 5 years of age (%)**				
Neonatal causes	18.3	27.4	25.9	26.2
Diarrhoeal diseases	18.8	15.6	18.3	16.6
Malaria	20.3	13.6	16.9	17.5
Pneumonia	23.3	18.6	23.9	21.1
Measles	3.4	2.5	6.1	4.3
HIV/AIDS	4.0	6.2	1.6	6.8
Other	11.9	16	7.3	7.5

* Source: World Health Statistics 2006

†WHO African region comprises 46 countries in sub-Saharan Africa including Algeria and Mauritania (African countries outside WHO/AFRO region are Western Sahara, Morocco, Tunisia, Libya, Egypt, Sudan, and Somalia).

### Study design

We conducted retrospective surveys of costs for integrating surveillance and response to the priority diseases adopted by each country (see Additional file 1, Appendix 1) at national, regional, and district surveillance offices as well as public health clinics, laboratories and pharmacies. We conducted one field test in Eritrea followed by full field studies in Burkina Faso and Mali. In Eritrea, the survey sites included the central Ministry of Health, the Anseba provincial office, and offices in the Haquaz district. In Burkina Faso, most IDSR activities were focused on the epidemic-prone diseases, with particular emphasis on detection and response to meningococcal meningitis. The data were obtained from four health regions (Bobo Dioulasso, Gaoua, Kaya, and Ouahigouya), 14 districts, and 20 primary health care centers. In Mali, the survey was conducted in three regions (Kayes, Mopti, and Sikasso), and included one district per region and one primary health care center per district. We consulted, in each country, with public health and disease surveillance officers to select sites that they considered representative of the national IDSR system.

We took the perspective of the government-funded health care system (i.e., we only recorded costs incurred by the governments and external partners). All cost data were recorded in local currency values and then converted into US dollar using the appropriate mean annual exchange rate. We used the general consumer price index from each country and a discount rate of 3% to adjust all costs into 2002 US dollars equivalent [22]. We also examined the effect on cost per capita estimate of using purchasing power parities (PPP) to convert national currencies into international dollars (PPP removes currency conversion problems due to fixed conversion rates that may not reflect actual relative costs) [22].

### Cost data

We collected data associated with all "health-related surveillance" (HRS) activities (i.e., all communicable and non-communicable diseases and risk factors, including the surveillance and response activities of the IDSR targeted diseases) from Burkina Faso and Eritrea for the years 2002 to 2005 and from Mali for the years 2000 to 2005. For each country, region/province and district, we obtained annual population data from the disease surveillance units. Program cost data were obtained from disease surveillance budget and program records, and from interviews with IDSR program coordinators and key public health staff. Whenever we found a difference between budget and reported expenditure, we used the reported expenditure. Aggregated pharmacy, clinical and medical records were collected using a structured questionnaire. The survey instrument (available from ) guided collection of data on all the resources used, including capital (one-time investment) and recurrent (on-going) items. The capital items included building infrastructure, vehicle, equipment (e.g., refrigerators, computers, etc.), and furniture (e.g., tables, chairs, etc.). The recurrent items included personnel (salaries and benefits of surveillance officers, data managers, physicians, nurses, etc.), rent (rent, utilities, operation, and maintenance), office and laboratory supplies, transportation, public awareness campaigns and short-term training. The questionnaire also collected information on other variables related to disease surveillance activities, such as length of use of buildings, vehicles or equipment per year, and resources provided through other activities and organizations.

### IDSR specific cost estimation

For each health structure level, all resources were grouped into the following major categories: personnel; transportation; office consumable goods; public awareness campaigns; drugs or treatment; laboratory supplies; and capital items (Additional file 1, Appendix 2). For each category, we identified the proportion of those cost data (such as staff workload or actual use of resources, if estimates or records were available) attributable to IDSR.

#### Personnel costs

When time keeping records were absent, we interviewed each staff member to estimate the breakdown of their time on all HRS, IDSR priority diseases, each IDSR activity (i.e., detection, notification, analysis, investigation, response, feedback, and support), and other ministry of health activities. We recorded the number of workers, their annual income, and the number of full time equivalents needed for administration or delivering of each HRS and IDSR activity. We then apportioned total personnel costs to each IDSR activity based on the ratio of personnel time allocated to that activity relative to all IDSR activities. We included fees of individual consultants hired for specialized services such as short-term training.

#### Transportation costs

We considered vehicles purchased for IDSR activities as capital items (see below). IDSR-related running costs for transporting personnel and patients, drugs, specimens, vaccines and other items, as a percentage of the total fuel and maintenance costs, were estimated based on the vehicle use-time per IDSR activity. When there were no data to apportion transport costs, we proportioned costs using the ratio of personnel time for IDSR to total personnel time for all HRS activities. We included rental vehicle and public transportation fees for IDSR-specific activities.

#### Office consumable costs

These included office supplies and materials, facilities and equipment maintenance, and utilities costs. Office consumable costs for IDSR, as a proportion of all HRS costs, were calculated using either the ratio of IDSR personnel time to all HRS personnel time, or actual amount of resources used for IDSR-activities (if the latter were available).

#### Public awareness campaign costs

We measured advertising, broadcasting and media costs for public campaigns and targeted social mobilization. IDSR costs were estimated as a proportion of total media health education costs using the ratio of IDSR personnel time to all HRS personnel time.

#### Treatment costs

These included all drugs and vaccines as well as other programmatic measures (e.g., treated bed nets) used in the line of controlling and preventing diseases included in the IDSR program. Total annual costs were calculated based on the procurement cost and the quantity of each specific product required for the treatment of diseases. We estimated IDSR costs using either the actual amount of resource or the ratio of IDSR personnel time to all HRS personnel time at the health facility (if the former were available).

#### Laboratory consumable costs

We estimated the costs of laboratory consumable materials and supplies (e.g., reagents, slides, gloves, test tubes, cotton wool swabs, blood culture bottles, aluminum foil, syringes, rapid diagnostic kits, etc) required for the purpose of various diagnostic tests for diseases included in the IDSR strategy.

#### Capital equipment costs

The costs of buildings, laboratory and office equipment and vehicles were depreciated at 3% annually over a 50-, 10-, and 5-year useful-life time horizon, respectively. We calculated the annualized cost using the following general equation:

$$\text{Annualized cost}=\text{K}\left[\frac{r{\left(1+r\right)}^{t}}{{\left(1+r\right)}^{t-1}}\right]$$

where *K* is the purchase price of the item, *r* represents the depreciation rate, and *t* is the useful-life-year. We assumed the scrap value of the capital items at the end of the useful life to be zero.

For equipment and vehicles, we apportioned out capital costs using the equipment and vehicle use-time (see above). For buildings, we proportioned capital costs using the ratio of IDSR personnel time to all HRS personnel time.

### Data analysis

We entered and analyzed the data in a spreadsheet (Microsoft Excel 5.0, Microsoft Corp., Seattle), calculating averages and standard deviations per resource category and per IDSR activity. We aggregated costs of all HRS and IDSR activities across all resource categories by health structure level. Using the estimated total costs for each province and district included in the study, and population estimates for each included province and district, we calculated average annual cost per capita per year for all HRS and IDSR activities. We then used these per capita costs and the annual population estimates to calculate the total annual national IDSR program cost in each country. We also compared the per capita surveillance costs to the per capita national health expenditures [23].

### Missing data

We encountered two types of missing data. The first category of missing data involved cost data for some building structures and equipment. For example, cost data were missing for approximately half of buildings in each country. The second category of missing data involved cost data for the laboratory testing and treatments from Burkina Faso. To fill in for the structure and equipment cost data, we used average cost data for similar structures and equipment at other sites (in the same country) as a proxy for the missing data. For example, when the information necessary to estimate the cost of a specific building was not available, we used the data for similar ministry buildings in the same locality or nearby health structures. For the missing cost data from Burkina Faso, we conducted two analyses: one by cost category (personnel, transport, office, etc.) excluding any cost categories for which we had no data and the other by extrapolating the relevant cost data from the other countries.

## Results

Table 3 summarizes the mean annual costs by resource categories at the region, district, and primary health care center level in the three countries. Detailed costs are shown in Additional file 1, Appendix 3. As expected, because of larger populations and types of IDSR activities, regional-level costs were greater in all categories than at the level of district and primary health care center. However, the cost of running IDSR at each site varied substantially by resource-type. Since disease surveillance requires trained staff, mean annual personnel costs were among the largest components of the region (10% to 47%) and district (16% to 44%) total IDSR costs in all three countries. Based on the results from Eritrea and Mali, we estimated that the laboratory and treatment costs ranged from 4% to 35% of the total IDSR cost in Burkina Faso. The proportion of the total IDSR cost due to treatment varied considerably (2% to 13%) by health structure in Eritrea and Mali. In general, the annualized capital costs constituted 2% to 13% of the total annual cost of IDSR in Eritrea, 6% to 12% in Mali, and 8% to 15% in Burkina Faso.

**Table 3:** Mean annual costs (in 2002 US $) of all health-related surveillance and IDSR per category of resources in Burkina Faso, Mali, and Eritrea

Health structure level	Cost category	Burkina Faso§		Mali		Eritrea	
				
		All health-related surveillance	IDSR	All health-related surveillance	IDSR	All health-related surveillance	IDSR
Region	Personnel	15,275	3,568	25,951	11,353	82,589	32,622
	Transport	13,015	4,771	18,226	7,292	4,137	3,309
	Office	13,102	5,471	31,362	10,889	67,032	27,643
	Media	1,664	238	4,515	1,481	0	0
	Treatment	55,964^§^	12,391^§^	14,007	3,594	30,789	3,506
	Laboratory	27,275^§^	5,032^§^	9,156	2,301	12,759	1,726
	Capital	11,271	4,429	8,368	2,663	8,026	1,114
							
District	Personnel	7,735	1,686	18,484	7,341	7,488	3,541
	Transport	10,712	2,159	16,519	2,233	5,490	1,098
	Office	7,855	1,807	5,642	1,718	7,141	5,358
	Media	527	116	677	169	0	0
	Treatment	13,571^§^	2,986^§^	3,409	369	2,029	350
	Laboratory	6,577^§^	1,209^§^	322	79	513	100
	Capital	4,318	826	6,301	2,032	5,561	1,540
							
Primary^¶ ^health	Personnel	1,839	478	2,752	728	1,974,579	191,584
care center	Transport	627	166	274	53	42,804	42,043
	Office	993	186	270	49	359,817	42,988
	Media	233	42	14	3	36,292	35,738
	Treatment	591^§^	131^§^	1632	182	756,914	123,547
	Laboratory	288^§^	53^§^	0	0	345,554	56,878
	Capital	624	184	909	167	119,475	16,204

§ In Burkina Faso, laboratory and treatment costs were calculated using the average annual per capita costs of laboratory and treatment for Eritrea and Mali

¶ In Eritrea, data were for the central Ministry level (primary health care center was not included in the study).

**Figure 2:**
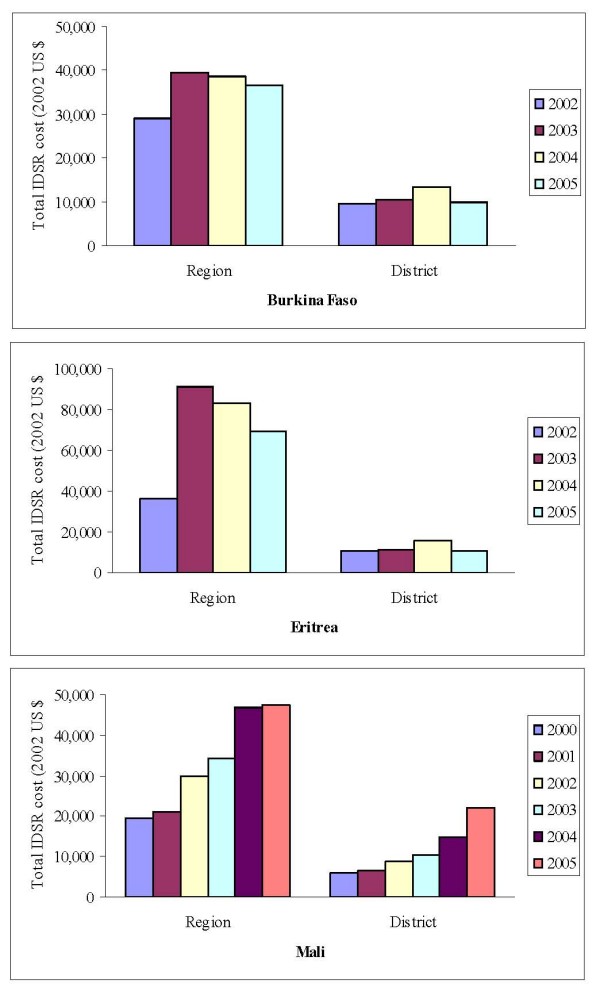
**Total IDSR cost per region and district in Burkina Faso^†^, Mali and Eritrea**. ^†^In Burkina Faso, total annual IDSR costs included costs extrapolated from the average per capita costs of laboratory and treatment for Eritrea and Mali.

The mean annual costs by health structure levels from the three countries surveyed are presented in Table 4. Eritrea had the highest total IDSR-related costs, and Burkina Faso had the lowest costs. The mean cost of IDSR, expressed as percentage of all HRS cost, also varied by health structure level (Table 4). In all three countries, the mean annual IDSR costs were 20% to 43% of the all HRS costs. In Burkina Faso and Eritrea, the highest costs ($39,419 and $91,362) of IDSR program per region occurred in 2003, and the highest costs ($13,297 and $15,781) per district in 2004 (Fig. 2). These were possibly associated with start-up costs of IDSR implementation at regional and district levels. By the end 2003, for example, Eritrea had completed training on IDSR in all the provinces; Burkina Faso had trained 18 national core trainers, 135 province supervisors, 110 laboratory technicians, and 1233 district and primary health care personnel; and Mali had trained only 406 health personnel from 28 districts of the four regions including Bamako.

**Table 4:** Mean annual costs* of IDSR strategy in comparison to all disease surveillance† systems in Burkina Faso, Mali, and Eritrea

Country	Health structure level	All health-related surveillance	IDSR	IDSR as % of all health-related surveillance
Burkina Faso¶				
	Region	137,566 *(18,231)*	35,899 *(4,746)*	26.1 *(5.51)*
	District	51,296 *(4,388)*	10,790 *(1,714)*	21.0 *(2.40)*
	Primary	5,196 *(965)*	1,240 *(161)*	23.9 *(2.92)*
Mali				
	Region	111,584 *(23,116)*	39,573 *(8,977)*	35.5 *(2.62)*
	District	51,354 *(27,864)*	13,941 *(5,892)*	27.1 *(2.91)*
	Primary health care center	5,851 *(1,699)*	1,181 *(780)*	20.2 *(7.90)*
Eritrea				
	Province	205,333 *(29,914)*	69,920 *(24,386)*	34.1 *(9.4)*
	District	28,220 *(4 411)*	11,985 *(2547)*	42.5 *(2.8)*

*All costs were converted to 2002 US dollar equivalent. Values in parenthesis are standard deviation from the means (2002 – 2005) of 4 health regions, 14 districts and 20 primary health care centers in Burkina Faso, 3 regions, 3 districts and 3 primary health care centers in Mali, and 1 province and 1 district in Eritrea.

† All health-related surveillance involves all communicable and non-communicable diseases and health risk factors, including the IDSR targeted diseases and conditions listed in Additional file 1, appendix 1.

¶In Burkina Faso, total costs included costs extrapolated from the average per capita costs of laboratory and treatment costs for Eritrea and Mali (see Table 2). Without the laboratory and treatment costs, the mean annual all disease surveillance and IDSR program costs were 54,327 and 18,476, 31,147 and 6,594, and 4,316 and 1,056 per region, district, and primary health care center level, respectively.

Costs disaggregated into IDSR activities are shown in Table 5. Detailed costs by year per IDSR activity are presented in Additional file 1, Appendix 4. As expected, the surveillance activities (e.g., detection, report, and analysis) that are carried out on a routine basis absorbed more resources than the support activities (e.g., evaluation and monitoring). Outbreak investigation and treatment of confirmed cases also constituted a substantial component (23% to 67%) of the total IDSR cost. When evaluating costs allocation at district level, detection of cases cost 21% to 40% of total IDSR costs, while outbreak investigation and verification accounted for only 2% to 18% of total IDSR cost. However, primary health care centers spent 8% to 67% of the total IDSR resources on detection and treatment of disease cases.

**Table 5:** Mean annual costs (standard deviation) by IDSR activity* per health structure levels in Burkina Faso, Eritrea and Mali: 2002 – 2005

Country	Health structure	Surveillance activities				Response activities		Support activities	
				
		Detection	Report	Analysis	Feedback	Investigation^§^	Treatment	Evaluation	Others†
**Burkina Faso¶**	Region	3,257 (648)	5,158 (1,567)	2,611 (791)	2,410 (784)	7,647 (1,216)	12,391 (1,331)	2,161 (420)	265 (85)
	District	2,248 (917)	1,485 (417)	821 (271)	601 (142)	1,971 (351)	2,924 (309)	721 (46)	143 (60)
	Primary health care center	305 (47)	238 (12)	146 (64)	130 (16)	164 (14)	129 (14)	57 (37)	69 (71)
									
**Eritrea**	Central	120,260 (33,084)	52,510 (26,766)	17,536 (8,013)	14,539 (4,660)	54,804 (6,442)	178,760 (50,404)	29,998 (11,216)	40,844 (17,325)
	Province	15,323 (8,064)	17,081 (6,739)	5,953 (2,596)	2,901 (1,035)	7,427 (3,507)	10,137 (2,670)	2,402 (629)	8,697 (2,842)
	District	3,702 (721)	2,495 (671)	1,225 (171)	542 (52)	1,059 (206)	1,660 (269)	40 (4)	1,263 (742)
									
**Mali**	Region	441 (180)	3,989 (2,183)	1,557 (216)	2,736 (815)	3,877 (216)	12,430 (2,961)	515 (520)	16,348 (4,791)
	District	5,629 (1,940)	1,268 (456)	723 (861)	395 (171)	334 (70)	4,002 (2,077)	212 (160)	1,378 (648)
	Primary health care center	98 77	57 (58)	1 (1)	0 (0)	7 (14)	794 (573)	0 (0)	224 (109)

* Costs per IDSR activity were converted to 2002 US dollar equivalent. To calculate the cost of each resource per IDSR activity, we multiplied the estimated total IDSR cost of that resource by the proportion of personnel time (or actual amount of resource) allocated to that activity relative to all IDSR activities.

§ Investigation, verification and laboratory confirmation of suspected cases.

† Other support activities include training, supervision, communication and coordination.

¶Laboratory and treatment costs in Burkina Faso were extrapolated from the average annual per capita costs of laboratory and treatment for Eritrea and Mali and the average population per health structure level in Burkina Faso (see Table 2)

The mean cost in Eritrea for an integrated surveillance system per capita was $0.16, which was 4 and 8 times larger than the $0.04 and $0.02 per capita recorded in Burkina Faso and Mali, respectively (Table 6). When we estimated costs using PPP, the mean cost per capita of IDSR for Eritrea was $0.87 and $0.06 for Mali (14 times larger). Eritrea's higher costs were possibly tied to post-war rebuilding of the national infrastructure, including the health system (see Additional file 1, appendix 5 for detailed IDSR budget in Eritrea). In Burkina Faso, we did not collect laboratory and treatment data. Instead, we extrapolated the costs from average annual cost of laboratory supplies and treatment for Eritrea and Mali. Without the laboratory and treatment costs, the mean annual per capita cost of IDSR in Burkina Faso and Mali was $0.02 compared to $0.13 in Eritrea. Using annual population estimates (Table 2) and the average per capita costs (Table 6), we estimated that the total annual national integrated surveillance program cost $476,208 in Burkina Faso, $690,957 in Eritrea and $270,360 in Mali. These accounted for 24% to 40% of the total HRS costs in all three countries. The per capita costs spent on all IDSR activities represented 3.2% (in the case of Eritrea) or less (in the case of Burkina Faso and Mali) of the total per capita government health budget.

**Table 6:** Mean annual per capita disease surveillance* and total health care costs (standard deviation): Burkina Faso, Eritrea, and Mali

Country	IDSR strategy				All health-related surveillance	National expenditure on health‡	
			
	Surveillance activities#	Response activities¶	Support activities§	Total IDSR†		Total expenditure	Government only
	Per capita cost (2002 US $)						
Burkina Faso	0.014	0.020	0.002	0.036	0.136	15.86	6.86
	(0.004)	(0.001)	(0.0004)	(0.005)	(0.023)	(3.93)	(2.27)
							
Eritrea	0.086	0.049	0.021	0.157	0.66	8.14	4.86
	(0.034)	(0.023)	(0.008)	(0.041)	(0.44)	(0.69)	(0.90)
							
Mali	0.005	0.008	0.007	0.020	0.05	13.60	7.00
	(0.001)	(0.0003)	(0.004)	(0.008)	(0.01)	(3.21)	(2.34)

* Cost per capita was calculated using the annual population size and all health-related (i.e., all communicable and non-communicable diseases and risk conditions) surveillance and IDSR costs for each health region/province and district included in the study in each country from 2000 to 2005. Total number of regions and districts surveyed each year in Burkina Faso, Mali and Eritrea was 18, 6 and 2, respectively.

# Surveillance activities include detection, report, analysis and feedback

¶Response activities include field investigation and laboratory confirmation of suspected cases and treatment of confirmed cases. In Burkina Faso, laboratory and treatment costs were calculated using the average annual per capita costs of laboratory and treatment for Eritrea and Mali and the population size of the health structure in Burkina Faso (see Table 2). Without laboratory and treatment costs, the annual costs (std. dev) per capita of IDSR and total national disease surveillance were $0.019 (0.005) and $0.055 (0.013), respectively.

§Support activities include training, supervision, evaluation, communication and coordination.

† Costs shown were converted using official exchange rate. When costs were converted using the purchasing parity power (PPP), the mean cost for Eritrea was $0.87 (0.34) and for Mali $0.06 (0.03).

‡ Source: National Health Accounts

## Discussion

IDSR attempts to integrate multiple, competing vertical systems in order to use surveillance and response-related resources more efficiently and reduce duplication of effort, especially at district and primary health care center levels [20,21]. In this study, we measured the incremental costs of setting-up and implementing an integrated surveillance and response strategy in Burkina Faso, Eritrea and Mali. In each country, the cost of IDSR was dependent on the health structure level. The district and primary health care center levels had much lower costs, as they usually had only lower cadre health workers and disease surveillance officers to provide services. A full understanding of the between-country differences in per capita costs of IDSR will require further study. As shown when we used PPP to convert local costs into US dollars, difference exchange rates may alter the degree of differences between countries.

The study's main limitation is the potential inaccuracy when we apportioned total cumulative surveillance activities cost (e.g., personnel time and building, equipment and vehicle use-time) to IDSR-specific activities. Log books of time and expenses did not provide the level of details needed to accurately divide out costs between IDSR and other surveillance and public health activities. As explained, we used the proportion of personnel time given to IDSR to proportion other costs. Furthermore, our retrospective survey may not have fully captured all costs due to the limitations of data records (e.g., no personnel time keeping records and the usual recall bias) in these countries. It is also possible that our data collection methods missed some surveillance-related expenditures. This is because, in Africa, donors often support specific public health projects (such as surveillance for a specific disease) that run parallel to the national public health system. Such projects often have a distinct identity (i.e., names and logos), and may even have staff paid directly by donor funds. Public health staff may not consider such projects part of the general public health system when enumerating costs associated with surveillance and IDSR. Another limitation of this study is the reliance on expenditure data, which may be weakened by over- and under-estimation and incomplete recording and do not reflect the whole economic cost. Further, indirect costs and productivity losses were not incorporated. Moreover, our estimate of IDSR cost based on 4-year data may be higher than when a longer term perspective is taken due to non-recurring start up costs. Absolute difference in cost per capita will depend upon the exchange rates used.

This study focused only the cost of resources accrued to IDSR activities and not the impact on the indicators used by the countries to monitor and evaluate their progress with their IDSR activities. In Eritrea, for example, the completeness of reporting case-based data from the health care center to the next high level increased from 50% in 2000 to 93% by the end of 2003. In Burkina Faso, the timeliness of surveillance reporting, especially data on epidemic-prone diseases, increased from 71% in 2000 to 99% by the end of 2004. Although Mali had also achieved the 80% target for these progress indicators, the transmission of complete data on time (83%) in 2005 was lower than that in Burkina Faso and Eritrea.

There are few studies on the costs of disease surveillance, and those are often not directly comparable to our study [16,24]. For example, John et al. (1998) measured the cost of emerging childhood vaccine-preventable diseases in India [16]. They found surveillance cost $0.01 per capita (1998 US $), which is approximately equal to the costs we measured in Burkina Faso and Mali (Table 6). However, the program in India only included childhood vaccine-preventable diseases, while the IDSR system includes not only childhood and adult vaccine-preventable diseases but also epidemic-prone diseases and endemic epidemics such as HIV/AIDS, malaria, TB, childhood diarrhea and acute respiratory infections. We can, therefore, consider IDSR a low cost public health system although the benefits, such as cases prevented, due to the IDSR program have yet to be quantified.

## Competing interests

The authors declare that they have no competing interests.

## Authors' contributions

ZCS, MIM, HNP conceived, carried out the study, analyzed the data, and drafted the manuscript. UA, MS, KT, and SOK each participated in the organization and coordination of the field data collection. NRM, TO, WA and IS participated in the design and coordination of the study. All authors read and approved the final manuscript.

## Supplementary Material

Additional file 1 **Appendix _ Cost Analysis of IDSR.** Appendix 1. List of IDSR priority diseases and diseases of public health importance weekly or monthly reported in Burkina, Eritrea, and Mali during the study period. Appendix 2. The following table includes the IDSR functions (Identify, Report, Analyze, Investigate, Respond, Feedback, Evaluate, etc.) and the general categories of implementation inputs (Personnel, Transport, Office Supplies, Public awareness Campaign, laboratory and treatment supplies, and Capital items). The table provides a few examples of specific costs related to the function and inputs. Many cells are left blank to illustrate that each country and health structure level (Central, Province/Region, district, and primary health center) will have different demands for costs. Appendix 3. Total annual cost (2002 US dollar equivalent) by year of each category of resources allocated for all disease surveillance † and IDSR-only activities in Burkina Faso, Eritrea, and Mali. Appendix 4. Mean annual costs (in 2002 US $) by year per IDSR-only activities in Burkina Faso, Eritrea, and Mali. Appendix 5. Budget allocated for IDSR implementation by year and estimated annual cost of national IDSR activities in Eritrea. Click here for file
